# Institutional Needs

**Published:** 1914-02-21

**Authors:** 


### Institutional Needs.

### ALIMENTARY ENZYMES.

(Benger's Food, Ltd., Manchester.)

The real value of a proprietary food or drug is usually in inverse ratio to the amount of mystery surrounding its composition and preparation. Ever since the late Mr. Benger became associated with Sir William Roberts, thirty-five years ago, and began the manufacture of the various foods and ferments now so widely used, there has been a degree of candour and frankness about all the productions of his firm which has been a splendid guarantee of their rational scientific basis. There has been nothing secret about the celebrated liquor pancreaticus or any of the other enzymes issued by the firm, and the composition of the well-known food is equally public property. Lately, Messrs. Benger have gone a step further by issuing an elaborate and accurate compendium of the latest researches on alimentary enzymes in book form. This interesting volume will enhance the confidence of the medical profession and the institutional world in all that issues from these professional and enlightened manufacturing scientists.

### ROYAL WARRANT FOR A. WULFING AND CO.

The latest of the many honours bestowed on Messrs. A. Wulfing and Co. at the professional congresses and exhibitions is the Royal Warrant which the King of Spain has given to Messrs. Wulfing for Sanatogen, Albulactin, and Formamint. The bestowal of this Warrant gains additional interest from the report that Albulactin is used in the Royal nursery for their Majesties' infants.

